# Sex and Caste-Specific Variation in Compound Eye Morphology of Five Honeybee Species

**DOI:** 10.1371/journal.pone.0057702

**Published:** 2013-02-27

**Authors:** Martin Streinzer, Axel Brockmann, Narayanappa Nagaraja, Johannes Spaethe

**Affiliations:** 1 Department of Behavioral Physiology and Sociobiology, Biozentrum, University of Würzburg, Würzburg, Germany; 2 Department of Evolutionary Biology, Faculty of Life Sciences, University of Vienna, Vienna, Austria; 3 National Centre for Biological Sciences, Tata Institute of Fundamental Research, Bangalore, India; 4 University Grants Commission, Academic Staff College, Bangalore University, Bangalore, India; Lund University, Sweden

## Abstract

Ranging from dwarfs to giants, the species of honeybees show remarkable differences in body size that have placed evolutionary constrains on the size of sensory organs and the brain. Colonies comprise three adult phenotypes, drones and two female castes, the reproductive queen and sterile workers. The phenotypes differ with respect to tasks and thus selection pressures which additionally constrain the shape of sensory systems. In a first step to explore the variability and interaction between species size-limitations and sex and caste-specific selection pressures in sensory and neural structures in honeybees, we compared eye size, ommatidia number and distribution of facet lens diameters in drones, queens and workers of five species (*Apis andreniformis*, *A. florea*, *A. dorsata*, *A. mellifera*, *A. cerana*). In these species, male and female eyes show a consistent sex-specific organization with respect to eye size and regional specialization of facet diameters. Drones possess distinctly enlarged eyes with large dorsal facets. Aside from these general patterns, we found signs of unique adaptations in eyes of *A. florea* and *A. dorsata* drones. In both species, drone eyes are disproportionately enlarged. In *A. dorsata* the increased eye size results from enlarged facets, a likely adaptation to crepuscular mating flights. In contrast, the relative enlargement of *A. florea* drone eyes results from an increase in ommatidia number, suggesting strong selection for high spatial resolution. Comparison of eye morphology and published mating flight times indicates a correlation between overall light sensitivity and species-specific mating flight times. The correlation suggests an important role of ambient light intensities in the regulation of species-specific mating flight times and the evolution of the visual system. Our study further deepens insights into visual adaptations within the genus *Apis* and opens up future perspectives for research to better understand the timing mechanisms and sensory physiology of mating related signals.

## Introduction

Honeybee colonies comprise three adult phenotypes, males (drones), reproductive females (queens) and sterile females (workers). Drones, queens and workers differ in reproductive organs, as well as in the morphology of mouthparts, flight musculature, number of glands, sensory systems and structural organization of brains [1]–[5]. Morphological and physiological differences correlate with behavioral differences and result from different natural and sexual selection pressures.

The virgin queen leaves the colony for mating flights; after mating, she remains in the hive and lays eggs for most of her life [1], [6]. The workers perform all tasks necessary to maintain the colony, e.g. brood care, foraging, and colony defense. Corresponding to this division of labor, queens show reductions in many morphological traits, e.g. mouthparts, pollen collecting structures, olfactory system and brain size [1], [4]. In contrast, drones engage neither in social nor foraging tasks; they serve predominantly for reproduction, i.e. searching for and mating with queens. Drones show enlarged and elaborated olfactory [2], [7], [8] and visual systems [9], as well as flight musculature that is adapted for fast pursuit flights [10].

Body size is considered the most characteristic morphological difference among honeybee species [11], [12]. Presumably, body size affects all sex and caste-specific morphological traits and, most importantly, sensory organs, the brain and motor system [10], [13], [14]. Body size scaling in bumblebees, for instance, results in more sensitive olfactory systems [14] and more acute and sensitive visual systems [13], [15] in larger bodied individuals, enabling foraging activities at lower light levels [13]. Large body size is further considered as an important pre-requisite for the behavioral transition to crepuscular activity [16].

Honeybees possess apposition compound eyes that consist of several thousand optically isolated ommatidia. Despite the general limitations given by the eye design, this eye type is well suited for orientation and object detection in bright daylight. Studies in sweat and carpenter bees show that, with some modification, the apposition compound eyes also enable reasonable visual orientation during night [17]–[20]. Eye design affects ecological success of a species, e.g. via improved flower detection capabilities in females and improved mate detection in males. It also sets an important boundary to the specific timeframe in which the animal is able to operate [20]. Numerous studies in Hymenoptera (e.g. ants [21], [22], bees [13], [17]–[19], [23], [24] and wasps [25]) document the relation between the structure of the visual system and the specific light environment in which the animal is active. In a recent study, Somanathan et al. [24] document visual adaptations of honeybee workers in three Asian species and the Western honeybee and discuss the implications of the eye design in the context of photic niche utilization for foraging. Temporal niche partitioning in honeybees is further important in the context of mating. Due to their common behavioral pattern of long range sex-pheromone attraction by a similar odor bouquet and short range visual chasing, geographically co-occurring species are forced to temporally separate mating times [11].

As an initial step to explore the variability of sensory and neural structures and the interaction between size limitations and sex and caste-specific selection pressures (e.g. selection on fecundity in queens, efficient foraging in workers and mate detection in drones) in *Apis*, we studied the periphery of the visual system. We investigated all sexes and castes of five species (*Apis andreniformis, A. florea, A. dorsata*, *A. mellifera* and *A. cerana)*, to contribute to our knowledge of caste and species-specific visual systems in the genus [17], [26]. We compared number and arrangement of ommatidia and facet lens diameters in the compound eyes and ocellar size. In particular, we asked whether and how eye size, ommatidia number and facet size correlate with body size and differ among sexes and castes. We hypothesize that clear deviations from body size correlations indicate specific adaptations, either with respect to spatial resolution (ommatidia number) or light sensitivity (facet diameter), both of which are traded-off against each other in relation to the specific lifestyle of the animal.

## Materials and Methods

### Honeybee Specimens

We investigated queens, workers and drones of the two dwarf honeybee species (*A. andreniformis* Smith, 1858 and *A. florea* Fabricius, 1787), the giant honeybee (*A. dorsata* Fabricius, 1793), the Western honeybee (*A. mellifera* Linnaeus, 1758) and the Eastern honeybee *(A. cerana* Fabricius, 1793). Specimens were collected near Bangalore, India (Doddaballapur, 13°17'32"N, 77°32'35"E) between 2003 and 2012 (*A. florea*, *A. dorsata, A. cerana*), in Chiang Rai Province, Northern Thailand (Mae Sai, 20°25'60"N, 99°52'60"E; Mae Fang Luang, 19°52'25"N, 99°43'23"E) in 2011 (*A. andreniformis*), in Vienna, Austria (48°13'47"N, 16°21'32"E) and Würzburg, Germany (49°46'48"N, 9°58'25"E) between 2009 and 2011 (*A. mellifera carnica)* and obtained from the collection maintained at the bee research unit, Bremen, Germany (*A. florea,* obtained from Feyriz, Fars Province, Iran in 1991). Specimens were either pin-mounted or preserved in ethanol and pin-mounted prior to measurements. For each species a minimum of four males and workers and two queens, were examined (except for *A. dorsata*; see Table 1 for sample numbers in parentheses).

**Table 1:** Body and eye measurements of five honeybee species.

Species	Caste/Sex	Body size^1^	Eye length	Eye surface	Ommatidia	Facet diameter	Ocellus med.	Ocellus lat.
		mm	mm	mm^2^		µm	mm	mm
*Apis florea*	queen	3.1±0.1 (3)	2.1±0.1 (3)	1.9±0.0 (2)	4,036±54 (2)	24.9±0.3 (2)	0.27±0.02 (3)	0.26±0.02 (3)
	worker	2.0±0.0 (5)	1.8±0.0 (5)	1.5±0.0 (4)	4,394±29 (4)	22.1±0.3 (4)	0.20±0.00 (5)	0.20±0.00 (5)
	drone	3.1±0.1 (5)	3.2±0.1 (5)	8.1±0.3 (4)	9,434±334 (4)	38.0±0.5 (4)	0.32±0.01 (5)	0.28±0.01 (5)
*Apis andreniformis*	queen	2.9±0.0 (2)	2.0±0.0 (2)	1.6±0.0 (2)	3,965±93 (2)	24.1±0.1 (2)	0.24±0.00 (2)	0.23±0.01 (2)
	worker	1.8±0.0 (4)	1.6±0.0 (4)	1.3±0.0 (4)	3,851±110 (4)	21.6±0.3 (4)	0.19±0.01 (4)	0.18±0.01 (4)
	drone	3.2±0.1 (5)	2.8±0.0 (5)	5.5±0.2 (4)	7,351±225 (4)	34.4±0.2 (4)	0.29±0.01 (5)	0.26±0.01 (5)
*Apis dorsata*	queen	4.3 (1)	2.9 (1)	4.1 (1)	4,479 (1)	34.7 (1)	0.38 (1)	0.40 (1)
	worker	3.1±0.0 (8)	2.9±0.0 (8)	4.1±0.2 (5)	5,974±112 (4)	30.8±0.7 (5)	0.40±0.02 (8)	0.37±0.02 (8)
	drone	3.8±0.1 (3)	3.6±0.1 (3)	10.7±0.7 (3)	8,383±463 (3)	46.3±1.0 (3)	0.40±0.00 (3)	0.34±0.01 (3)
*Apis mellifera*	queen	3.5±0.1 (4)	2.4±0.1 (4)	2.2±0.0 (2)	4,460±55 (2)	26.1±0.2 (2)	0.30±0.01 (4)	0.30±0.01 (4)
	worker	2.9±0.0 (6)	2.4±0.1 (5)	2.5±0.1 (4)	5,375±143 (4)	25.2±0.3 (3)	0.30±0.01 (5)	0.28±0.01 (5)
	drone	4.3±0.1 (5)	3.6±0.1 (5)	9.4±0.4 (4)	9,993±483 (4)	40.1±0.7 (4)	0.36±0.02 (5)	0.34±0.02 (5)
*Apis cerana*	queen	3.2±0.1 (5)	2.1±0.1 (5)	1.8±0.1 (3)	3,582±106 (3)	25.9±0.3 (3)	0.27±0.01 (5)	0.26±0.01 (5)
	worker	2.6±0.1 (8)	2.1±0.0 (8)	2.3±0.0 (5)	4,921±88 (4)	25.4±0.1 (5)	0.25±0.01 (8)	0.23±0.01 (8)
	drone	3.2±0.1 (4)	2.8±0.1 (4)	5.9±0.3 (4)	7,994±167 (4)	35.8±1.1 (2)	0.30±0.01 (4)	0.26±0.00 (4)

Measured parameters are given as means±std.dev. Sample size is indicated in parentheses. ^1^Body size is expressed as the distance between the wing bases (intertegulae span).

### Eye and Body Size Measurements

Size measurements of thorax, compound eyes and ocelli were performed on digital photographs using ImageJ (National Institute of Mental Health, Bethesda Maryland, USA). Photographs were taken with stereomicroscopes (Nikon SMZ-U equipped with DS-Fi1, Tokyo, Japan and Leica EZ4D, Leica Microsystems, Wetzlar, Germany) at different magnifications (4–16×). Size measurements were calibrated with respect to photographs of an object micrometer at the same magnification. As a measure of body size we used intertegulae span, which was previously shown to be an appropriate estimate of body size in bees [15], [27]. Eye length was measured as the longest linear measure across the eye from a frontal view. Eye surface area measurements were performed on eye surface replicas made of nail polish [26]. Replicas were photographed using light microscopes (Nikon Labophot equipped with DS-Fi1 and Zeiss Axiophot, Zeiss Germany equipped with Spot Insight Color, Diagnostic Instruments Inc., USA) at 100–400× magnifications in overlapping sections and subsequently stitched in Adobe Photoshop CS2 (Adobe Systems, San Jose, CA, USA). The eye surface was then measured by tracing the outlines in ImageJ. Measurements of the ocelli were performed as the longest linear measure across the median and the left lateral ocellus.

### Ommatidia Measurements

Ommatidia number was determined by manually marking all facet imprints of the eye replica in ImageJ. To measure the facet diameter, a row of 5 ommatidia in all three axes (x, y and z) was measured in ImageJ. We then calculated the mean diameter of a single ommatidium [13]. Measurements were performed on the largest facets. Additionally, eye maps were created to illustrate facet diameter distribution over the entire eye surface. ImageJ, Meshlab (Visual Computing Lab - ISRI - CNR, http://meshlab.sourceforge.net/) and CorelDraw X5 (Corel Corporation) was used to create the maps. In brief, ommatidia diameters were estimated from the distance between neighboring ommatidia centers. For visualization, ommatidia diameters across the eye surface were color coded.

### Mating Flight Activity

In addition to the size measurements, we analyzed published records of drone and queen flight activity of all investigated species. Reported flight times were corrected for solar azimuth differences according to the procedure employed by Otis et al. [28] when such a correction was not performed in the original study. For observations over several days we only report the total range of drone flight activity (e.g. [29]), and we aimed to avoid pseudo-replication from subsequent citations of the same original data set. Ambient light intensity is a function of solar elevation and not only depends on the time of the day but also on geographic latitude and time of the year. To transform daytime records of mating flight times to solar elevation information, we calculated the range of solar elevation for the flight period of all studies that reported location and date, using equations provided by the NOAA (U.S. Department of Commerce).

### Statistics

Body and eye parameters were compared between and within species with a Kruskal-Wallis H test. All P-values below the 5%-level were considered to be statistically significant. Statistical analyses were performed with Statistica 10 (StatSoft Inc., OK, USA).

## Results

### Body and Eye Size

**Figure 1:**
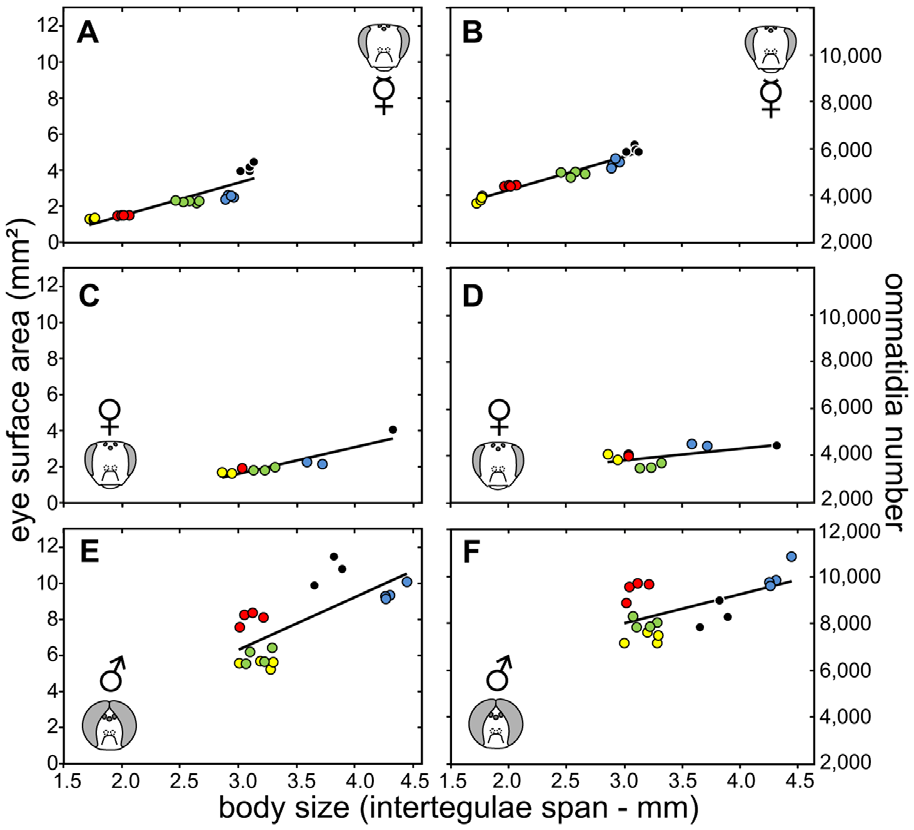
Morphological measurements of eye parameters in five honeybee species. Eye surface area (left panel) and ommatidia number (right panel) measured in workers (A, B), queens (C, D) and drones (E, F) of the Western and four Asian honeybee species. Species are indicated by color (*A. andreniformis* – yellow, *A. florea* – red, *A. dorsata* – black, *A. mellifera* – blue, *A. cerana* – green). Each circle represents one measured individual (see Table 1 for sample sizes). Trend lines are based on all measured specimens (A: y = 1.82x−2.15, R^2^ = 0.79; B: y = 1,421.66 x +1,399.65, R^2^ = 0.94; C: y = 1.46x −2.75, R^2^ = 0.81; D: y = 514.11x +2,312.18, R^2^ = 0.38; E: y = 2.88x –2.29, R^2^ = 0.51; F: y = 1,250.49x +4,264.23, R^2^ = 0.34).

The five investigated honeybee species differ with respect to body and eye size. The largest variation is found in workers and the smallest in drones (Table 1). Body size (intertegulae span) differs significantly, both between castes and sexes within species (H*_andreniformis_*(2,11) = 8.6,p<0.05; H*_cerana_*(2,17) = 12.3,p<0.005; H*_dorsata_*(2,12) = 7.6,p<0.05; H*_florea_*(2,13) = 8.6,p<0.05; H*_mellifera_*(2,15) = 12.4,p<0.005) and within castes and sexes among species (H_workers_(4,31) = 28.6,p<0.005; H_queens_(4,15) = 12.2,p<0.05; H_drones_(4,22) = 16.5,p<0.005). In all species, queens and males are larger than workers, whereas the polarity of size differences between males and queens varies among species. Males are larger than queens in *A. andreniformis* and *A. mellifera*, smaller than queens in *A. dorsata*, and similar in body size in *A. florea* and *A. cerana* (Fig. 1, Table 1). Among drones, *A. mellifera* drones are larger than *A. dorsata* drones, and the drones of *A. andreniformis*, *A. florea* and *A. cerana* are similar in size. Our results are consistent with previous weight measurements performed on several honeybee species [11].

**Figure 2:**
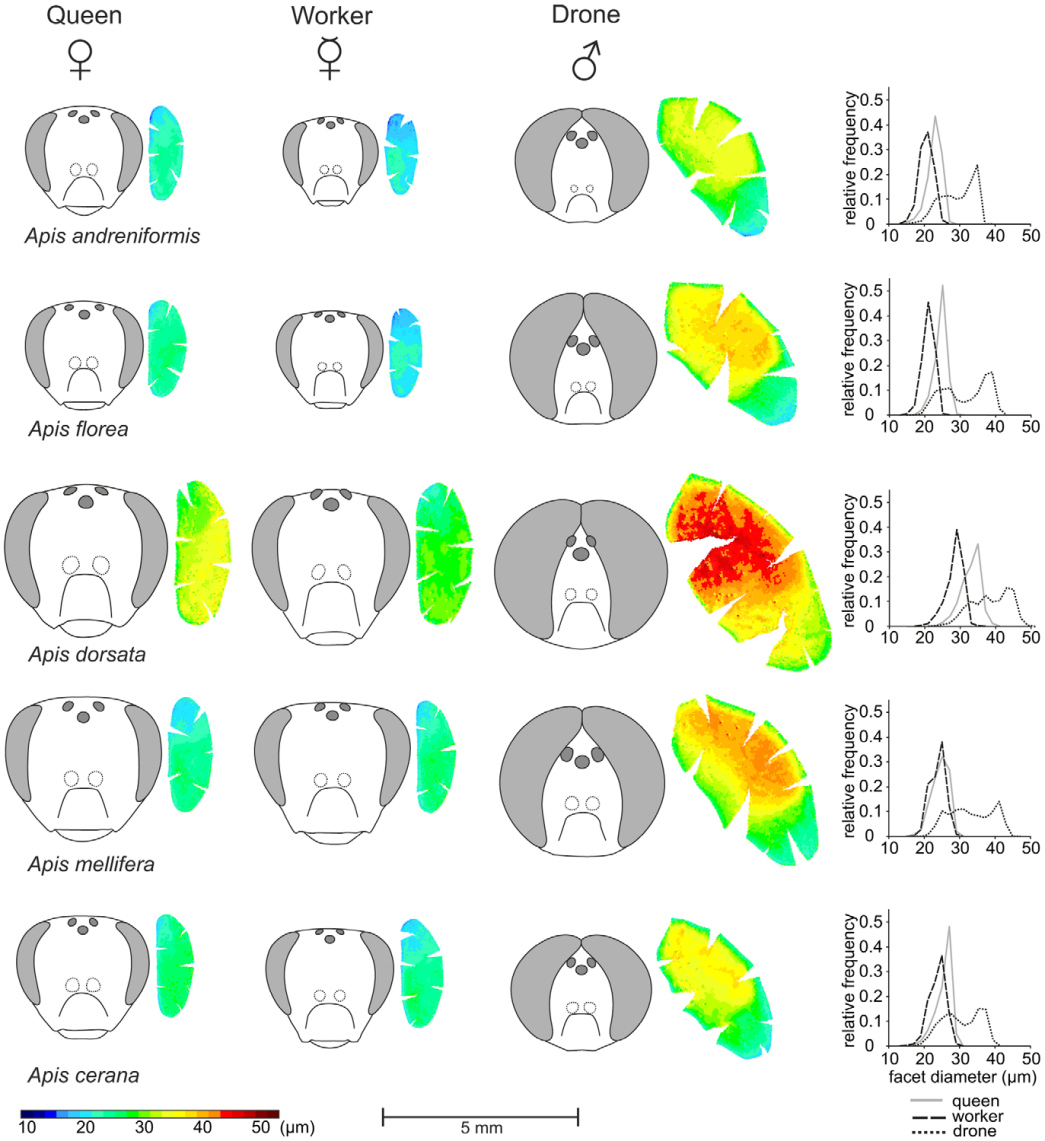
Facet size distribution in compound eyes. Eye maps illustrate eye size differences and facet size distribution between castes and sexes of the Western and four Asian honeybee species. Each circle represents one facet lens. Color indicates facet size (scale at the bottom). The largest facets in queens and workers are usually found in the fronto-ventral region of the eye. In drones, facet diameters are dorso-ventrally separated and the largest facets are found in the dorsal two-thirds of the eye. The eye maps are accompanied by line drawings of all individuals, which allow comparison of eye placement, eye size and ocellar size between species, sexes and castes (all to scale, scale bar 5 mm). Relative facet diameter frequencies are illustrated by histograms (right panel, bin width 2 µm) of one randomly selected queen (gray line), worker (dashed line) and drone (dotted line) from each species.

Eye size (eye surface area) differs significantly between sexes and castes in all species except for *A. dorsata* (H*_andreniformis_*(2,10) = 7.9,p<0.05; H*_cerana_*(2,12) = 9.7,p<0.01; H*_dorsata_*(2,9) = 5.4,p = 0.07; H*_florea_*(2,10) = 7.9,p<0.05; H*_mellifera_*(2,10) = 7.9,p<0.05). Within species, eye size is similar between workers and queens (Fig. 1, Fig. 2, Table 1). However, due to larger body size, queen eyes appear relatively smaller. In contrast, drones have much larger and differently shaped eyes (Fig. 2). Differences between drone and female eyes range from 2.6-fold in *A. dorsata* to 5.4-fold in *A. florea* (Fig. 1, Table 1). While eye size of queens and workers positively scales with body size in all species, drone eye size does not simply so. In particular, *A. florea* and *A. dorsata* drone eyes are disproportionally enlarged relative to body size compared with drones of the other species (Fig. 1, Table 1).

### Ommatidia Numbers and Facet Size Distribution

The number of ommatidia range from c. 3,500 in the queens of *A. cerana* to c. 11,000 in drones of the Western honeybee (Fig. 1, Table 1). In all species, except for *A. andreniformis*, queens possess the lowest, while drones possess the highest number of ommatidia (Table 1). The number of ommatidia differs significantly between castes and sexes in all species (Fig. 1, Table 1; H*_andreniformis_*(2,10) = 6.9,p<0.05; H*_cerana_*(2,9) = 7.0,p<0.05; H*_florea_*(2,10) = 7.9,p<0.05; H*_mellifera_*(2,10) = 7.9,p<0.05). For *A. dorsata* (H*_dorsata_*(2,8) = 5.8,p = 0.054) the p-value was marginal significant, due to the low sample size of queens (N = 1). Ommatidia numbers in worker and drone eyes differ among all species (H_workers_(4,20) = 18.3,p<0.005; H_drones_(4,17) = 14.3,p<0.01), whereas ommatidia number in queens do not differ among species (H_queens_(4,10) = 8.1,p = 0.09) despite strong variation in body size.

In apposition compound eyes facet diameters are usually not evenly distributed along the eye surface and the largest facets are commonly found in regions associated with high spatial acuity and light sensitivity [30]. In *Apis*, facet diameter frequency and distribution differs between sex and castes. Both female castes have a nearly Gaussian distribution of facet diameters and their largest facets are located in the fronto-ventral region of the eye (Fig. 2). In all species, except *A. mellifera*, queen eyes are composed of ommatidia with larger facet diameter compared with worker eyes. The largest difference in facet diameter between worker and queen eyes is found in *A. dorsata* (Fig. 2, Table 1). Drone eyes show a strong dorso-ventral regionalization, indicated by a steep transition in facet diameter (Fig. 2). The dorsal two thirds of the eye are equipped with large facets of which the largest are located in the dorso-lateral region. The ventral third is equipped with smaller facets that are similar in size to the largest facets in workers. The distinct dorso-ventral separation is also reflected in the diameter frequency distribution, which is flatter and shows more than one maximum in all species (Fig. 2).

### Ocelli

The three ocelli are located at the top of the head in queens and workers and frontal in drones (Fig. 2). Usually, the median ocellus is larger than the lateral ocelli (Table 1). Ocellus size differs significantly between castes and sexes in all species, both for the median (H*_andreniformis_*(2,11) = 8.6,p<0.05; H*_cerana_*(2,17) = 13.8,p<0.005; H*_dorsata_*(2,12) = 2.6,p = 0.28; H*_florea_*(2,13) = 10.6,p<0.01; H*_mellifera_*(2,14) = 9.6,p<0.01), and the lateral ocellus (H*_andreniformis_*(2,11) = 8.6,p<0.05; H*_cerana_*(2,17) = 12.4,p<0.005; H*_dorsata_*(2,12) = 7.3,p<0.05; H*_florea_*(2,13) = 9.5,p<0.01; H*_mellifera_*(2,14) = 8.9,p<0.05), with the exception of the median ocellus in *A. dorsata*. The largest ocelli are found in *A. dorsata* and the smallest in workers of the dwarf honeybees *A. andreniformis* and *A. florea*. In general, ocelli are larger in queens than in workers of the small species (*A. andreniformis, A. florea, A. cerana*), similar in size in *A. mellifera*, and smaller in *A. dorsata*. In drones, ocellus diameters show the same trend as facet size; *A. dorsata* drones have the largest, followed by *A. mellifera*. Ocelli in *A. florea* are larger than in the similarly sized drones of *A. andreniformis* and *A. cerana* (Table 1).

## Discussion

Our study documents sex and caste-specific variation in the compound eyes of five honeybee species. In queens and workers, eye size, ommatidia number, facet diameter and ocellus size positively correlate with body size among the species. Although queens are larger, queen and worker eyes are of similar size, but worker eyes on average comprise a higher number of ommatidia with smaller facets. Compared with the female castes, drones of all species show enlarged and highly modified compound eyes but drone eye size does not simply correlate with body size. Particularly, drones of the dwarf honeybee *A. florea* have disproportionately enlarged eyes in relation to body size and exhibit more ommatidia than drone eyes of the giant honeybee *A. dorsata* and almost as many ommatidia as drone eyes of the Western honeybee, *A. mellifera* (Figs. 1, 2; Table 1). Overall, the findings of our study indicate a greater variability in the design of drone visual systems than previously assumed and this variability is probably the result of the interaction of species-specific body size limitations and sex-specific selection pressures.

### Female Eye Morphology and Female Behavior

In all species, eye size is similar between queens and workers, but queens usually possess less yet larger ommatidia (Fig. 1). Queens spend most of their lives in the colony where vision plays a minor role. The few occasions when they leave the hive (mating flights, swarming, absconding and migration) certainly require good spatial vision; however, the demands for visual acuity are likely less strong than for workers, which need to detect and identify flowers and orient themselves during foraging flights. Additionally, we found that the compound eyes of queens from the open nesting honeybee species (*A. andreniformis*, *A. florea* and *A. dorsata*) have relatively enlarged facets compared with workers, while such an enlargement is only marginal (*A. cerana*) or absent (*A. mellifera*) in the cavity nesting species. The two cavity nesting species are closely related [31], yet we do not know whether the smaller relative queen facet size (i) is related to the predominant life inside the nest, (ii) constitutes a phylogenetic constraint or (iii) is a byproduct of other selection pressures (e.g. [32]).

The largest caste difference in facet diameters is found in *A. dorsata*. Queen facets are enlarged, at the expense of ommatidia number, suggesting that queens trade-off spatial resolution for increased light sensitivity, a likely adaptation for crepuscular mating activity. In the two dwarf honeybee species, the decrease in worker body (and thus eye) size is accompanied by a reduction of facet diameters, but not ommatidia number, suggesting that workers trade-off light sensitivity to retain spatial resolution, which is important in foraging tasks. The smaller facet diameters, however, may limit their foraging abilities during the twilight hours, compared with the species that possess larger facet diameters [13]. Somanathan et al. [24] recently investigated the worker eyes of four honeybee species. In concordance with our results, they found smaller eyes and facets in the dwarf species and enlarged facet and ocellar lenses in *A. dorsata*. Model calculation further suggest, that light sensitivity is highest in *A. dorsata* and lowest in *A. florea*, which correlates with the observed temporal foraging patterns ([24] and citations therein).

It must be noted that both spatial resolution and light sensitivity not only depend on the morphology of the peripheral optical system (ommatidia diameters and numbers), but also on the photoreceptor arrangement (interommatidial angles, rhabdom diameter) and potentially on neuronal computation strategies [18], [19], [23], [33]. For instance, model calculations by Somanathan et al. [24] suggested that *A. dorsata* has the lowest spatial resolution, despite having the highest number of ommatidia and that their light sensitivity is additionally increased due to larger rhabdom diameters. So far, detailed measurements of the interommatidial and acceptance angles, light sensitivity, and behavioral assessment of the spatial resolution, object detection threshold and light intensity threshold are lacking for all Asian honeybee species.

Our measured eye parameters of *A. florea* differ from the previously published data [24]. We suggest that regional intra-specific variation in body (and thus eye) size may account for the c. 900 more ommatidia we find in *A. florea* workers. We investigated *A. florea* workers and drones from Iran, while Somanathan et al. [24] collected workers in India. No subspecies are officially recognized in *A. florea*, but morphometric studies revealed the existence of several morphotypes and workers from Iranian populations are larger than workers from Indian populations (own measurements and [34]). Similarly, our measurements on *A. mellifera* differ from earlier reports (e.g. [17], [26]). The Western honeybee is widely distributed and comprises several distinct subspecies. For instance, three of the economically important subspecies, *A. m. mellifera*, *A. m. carnica* and *A. m. ligustica* differ with respect to body size (in this sequence, *A. m. mellifera* being the largest [35]). In addition, historical and regional differences in bee keeping management (e.g. the used foundation cell size) artificially constrain body size and may account for large intra-specific variation [36].

### Drone Eye Morphology and Drone Behavior

Several studies on eye morphology and drone chasing behavior in *A. mellifera* indicate that the drone compound eye is specialized to detect small moving objects against the bright blue sky [9], [37]–[41]. The eyes show distinct regional specializations, e.g. extremely enlarged facets located in the dorsal region of the eye [26]. Our study demonstrates a similar organization in drone eyes of all investigated honeybee species (Fig. 2). However, the drone compound eyes of *A. florea* and *A. dorsata* apparently have evolved specific adaptations. *A. florea* drones have much larger compound eyes than drones of the other dwarf honeybee *A. andreniformis*. Furthermore, relative to body size, their eyes are larger than in drones of all other honeybee species. The enlargement of the compound eyes is accompanied by an increase of ommatidia number suggesting a substantial increase in spatial resolution. In contrast, the large compound eyes of *A. dorsata* drones consist of a lower number of ommatidia compared with *A. florea* drones, but these ommatidia exhibit much larger facet lenses, suggesting a significant increase in light and, probably more important, contrast sensitivity [20].

**Figure 3:**
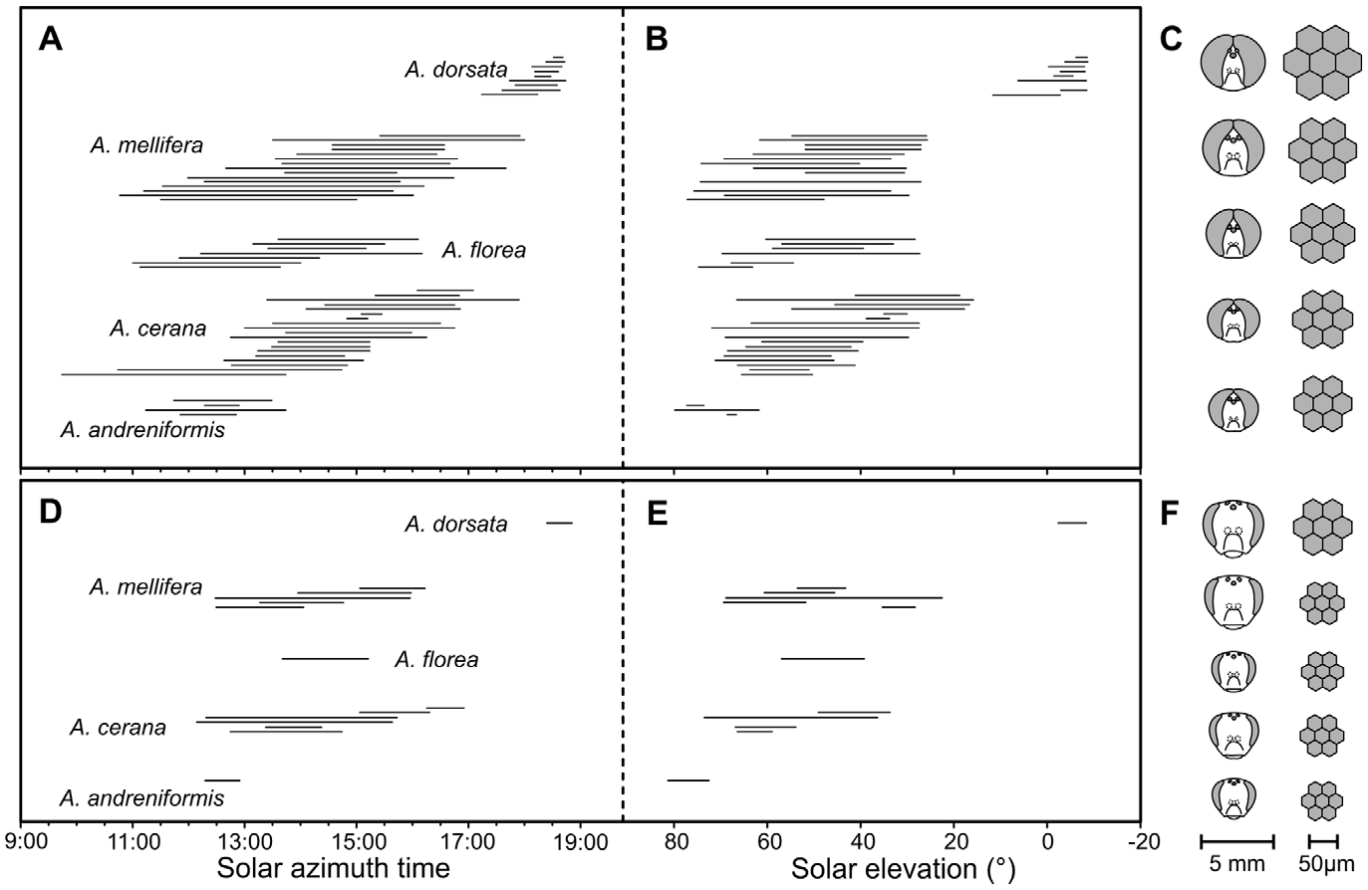
Species-specific mating flight activity. Drone (A, B, C) and queen (D, E, F) flight activity compiled from literature records [11], [29], [42], [45], [46], [49], [50], [56], [58]–[84]. (A, D) Temporal range and (B, E) corresponding solar elevation range of the flight time. When necessary, time was converted to solar azimuth time. For studies that did not report the date of observation, solar elevation could not be calculated (bars missing in B, E). (C, F) Graphical representation of the heads (left) and ommatidia diameters (right) for all species (scale bars below). Species are sorted in an ascending order of drone eye size, ommatidia and ocellar diameters.

In most honeybee species drone mating flights start around noon or early afternoon at times of highest solar elevation and light intensities and last until late afternoon (Fig. 3). Mating flight times in *A. dorsata* diverge from this pattern; they occur at sunset and are much shorter in duration. Daily onset and end of mating flights are strongly affected by changing ambient light and temperature conditions as well as the animal’s motivation [42]. In *A. dorsata,* facet and ocelli diameters resemble those of some strictly nocturnal bee species [17], [23], and may thus allow mating flights around sunset. *A. andreniformis* also seems to have a narrow mating flight period that is constrained to times of highest light intensities (Fig. 3). The findings of our study indicate a correlation between morphological characters of drone eyes and species-specific mating flight times, which reflects the importance of visual mate detection in honeybee mating behavior.

We find a similar correlation between facet and eye size and mating flight time in queens. Both sexes are active at the same time, ensuring successful meeting and mating. Queens, however, possess smaller facets than drones, suggesting that light levels alone may not account for the large facets in drone eyes. Drones face a tremendous challenge when detecting a fast moving queen from a distance. The visual acuity in the dorsal eye region is high [43] but probably even more importantly, the enlarged facet lenses give *Apis* drones an extremely high contrast sensitivity [39]. The large facet diameters of *A. dorsata* drones may thus be particularly important to maximize contrast sensitivity in low light environments that generally limit visual contrast [20]. The smaller facets and ocelli in *A. andreniformis* may constrain mating flights to high noon when light level and contrast ratios are sufficiently high (Fig. 2, Fig. 3). In the case of *A. dorsata*, body size might have been a pre-adaptation, which allowed a shift of mating flights to lower light levels [16]. Interestingly, the only report on drone mating flight times in *A. laboriosa*, the similar-sized Himalayan sister species of *A. dorsata*, indicated that mating flights start in early afternoon [44]. This shift is most likely a response to the harsh weather conditions at high elevations and a similar shift of mating flights is observed in the second Asian mountain honeybee *A. nuluensis* [45].

Based on our observations, we hypothesize that ambient light intensity is a major factor for the timing of honeybee mating flights. Although mating flight times exhibit a high degree of variability (Fig. 3, left panels), our calculations show that at least some of the variation is a result of differences in location [46] and time of year [42], [47] and can be explained by differences in solar elevation (Fig. 3, right panels). Similar to hypotheses on worker foraging behavior [48], we suggest that eye morphology and ambient light intensity define a species-specific timeframe for mating behavior. In the case of geographically co-occurring honeybee species, mating flight times can be shortened and shifted within this basic timeframe according to sensitivity of the visual system [49], [50]. Recent studies in Australian *Myrmecia* ants demonstrated that worker foraging activity is exclusively controlled by absolute light levels [21], and caste and species-specific activity schedules are determined by eye morphology [22]. However, at this time we cannot exclude the possibility that mating flight times in honeybees are also affected by other environmental parameters, such as ambient temperature and humidity, which correlate with light intensity. In addition, an impact of ambient light intensity levels on mating flight activity does not exclude that mating behavior is regulated by the circadian clock [51], [52]. The clock likely regulates physiological processes involved in mating behavior in anticipation of the actual mating flight. Future experiments should focus on the hitherto unknown proximate physiological and neuronal mechanisms that generate narrow and temporally separated mating flight periods in *Apis*. The potential to quickly adapt the mating timeframe in response to sympatric honeybee species in order to avoid inter-specific interference provides an avenue for future research on the function and evolution of the mechanisms that regulate the timing of mating flights.

### The Curious Case of *Apis florea* Drones

Characteristic of the honeybee mating behavior is that drones chase the queens [1]. Queens signal their presence by releasing their sex-pheromone, which triggers an upwind search by drones and also heightens their motivation to chase any small and dark object moving against the sky [39]. Although all *Apis* species are assumed to be highly sensitive to queen pheromone, differences in the number of olfactory sensilla suggest a unique exaggeration of the sex-pheromone specific olfactory system in *A. mellifera* [53], [54]. In contrast, *A. florea* drones have the lowest number and density of olfactory sensilla [53] and much smaller sex-pheromone processing macroglomeruli compared with *A. mellifera* drones [54]. Neural tissue maintenance and information processing are energetically costly and thus may be particularly prone to counter selection [55]. This limitation certainly affects the trade-off between different sensory systems, e.g. an enlarged visual system comes at the cost of a poorer olfactory system and *vice versa*. The current data on the olfactory and visual sensory systems in drone honeybees suggest that *A. mellifera* drones have specifically improved the sensitivity of their olfactory system in their evolution, whereas *A. florea* drones invested particularly in their visual system. We can only speculate about the ultimate causes for the differences among honeybee species. Brockmann and Brückner [53] suggested that low mate density may have promoted the evolution of a particularly sensitive olfactory system in *A. mellifera*. The question why drones of *A. florea* have evolved relatively enlarged eyes is of particular interest with respect to the fact that drones of the sister species *A. andreniformis* did not evolve similar traits. Almost nothing is known about the mating behavior and drone congregation areas of the latter two species [56]. Both species differ with respect to their preference for nesting and probably also mating areas [57]. However, current knowledge on mating related signals and cues and the specific tasks of the sensory system in honeybee mating behavior is limited and does not permit us to draw further conclusions about the evolution of their sensory systems.

### Conclusion and Future Perspective

Based on the assumption that body size differences interact with sex and caste-specific selection pressures, we compared four different characters of the visual system in drones, queens and workers of five honeybee species. This approach successfully identified common patterns of adaptation within castes and revealed distinct adaptations in the drone eyes of two species, *A. florea* and *A. dorsata*. In general, the variability among species seems to be caused by the interaction of different factors, such as body size limitations, different selection pressures (e.g. selection for mate detection, foraging efficiency and fecundity that are exclusive to drones, workers and queens, respectively), temporal activity pattern and different relative roles of the sensory systems (e.g. the importance of vision vs. olfaction during mate detection). In the future, it will be interesting to test whether these morphological differences are accompanied by differences in the behavioral responses to visual and olfactory signals.
